# Smooth Muscle-Alpha Actin R149C Pathogenic Variant Downregulates Integrin Recruitment at Cell-Matrix Adhesions and Decreases Cellular Contractility

**DOI:** 10.3390/ijms24119616

**Published:** 2023-06-01

**Authors:** Krishna R. Ojha, Hyoseon Kim, Samuel Padgham, Laura Hopkins, Robert J. Zamen, Abhijnan Chattopadhyay, Gang Han, Dianna M. Milewicz, Michael P. Massett, Andreea Trache

**Affiliations:** 1 Department of Medical Physiology, Texas A&M University Health Science Center, Bryan, TX 77807, USA; 2 Department of Kinesiology and Sport Management, Texas Tech University, Lubbock, TX 79409, USA; 3 Department of Epidemiology and Statistics, Texas A&M University Health Science Center, College Station, TX 77843, USA; 4 Department of Biomedical Engineering, Texas A&M University, College Station, TX 77843, USA; 5 Department of Internal Medicine, McGovern Medical School, University of Texas Health Science Center, Houston, TX 77030, USA

**Keywords:** integrins, actin, vascular smooth muscle cells, *Acta2^R149C/+^* mice

## Abstract

Thoracic aortic aneurysm is found in patients with *ACTA2* pathogenic variants. *ACTA2* missense variants are associated with impaired aortic smooth muscle cell (SMC) contraction. This study tested the hypothesis that the *Acta2^R149C/+^* variant alters actin isoform expression and decreases integrin recruitment, thus, reducing aortic contractility. Stress relaxation measurements in thoracic aortic rings showed two functional regimes with a reduction of stress relaxation in the aorta from *Acta2^R149C/+^* mice at low tension, but not at high tension values. Contractile responses to phenylephrine and potassium chloride were 50% lower in *Acta2^R149C/+^* mice than in wild-type (WT) mice. Additionally, SMC were immunofluorescently labeled for specific proteins and imaged by confocal or total internal reflection fluorescence microscopy. The quantification of protein fluorescence of *Acta2^R149C/+^* SMC showed a downregulation in smooth muscle α-actin (SMα-actin) and a compensatory upregulation of smooth muscle γ-actin (SMγ-actin) compared to WT cells. These results suggest that downregulation of SMα-actin leads to reduced SMC contractility, while upregulation of SMγ-actin may lead to increased SMC stiffness. Decreased α5β1 and α2β1 integrin recruitment at cell-matrix adhesions further reduce the ability of mutant cells to participate in cell-matrix crosstalk. Collectively, the results suggest that mutant *Acta2^R149C/+^* aortic SMC have reduced contractility and interaction with the matrix, which are potential long-term contributing factors to thoracic aortic aneurysms.

## 1. Introduction

Mechanical forces can regulate intracellular signaling pathways and substantially affect cell function in many biological processes. Extracellular mechanical signals are sensed by receptors at the cell surface and then transmitted intracellularly, contributing to cytoskeleton remodeling, and altering cell-matrix and cell-cell interaction with the ultimate goal of regulating vessel wall contractility and remodeling [1].

Actomyosin contractility is a key determinant of force development in vascular smooth muscle cells (SMC), while integrins serve as mechanotransducers that enable force transmission between cell and matrix. The loss of the *Acta2* gene encoding for smooth muscle alpha–actin, which is the predominant actin isoform in vascular smooth muscle, can affect vascular SMC function by reducing their contractile properties and ability to sense external mechanical stimuli [2]. *ACTA2* pathogenic variants predispose individuals to heritable thoracic aortic disease (HTAD) [3,4]. The most common recurrent missense variant in *ACTA2* is the alteration of arginine 149 to cysteine (R149C) [5], which is responsible for approximately a quarter of the patients with *ACTA2* mutations [6].

Cell-matrix adhesions connect vascular SMC to the matrix where mechanical stimuli are transferred back and forth across the plasma membrane through integrin receptors [7]. Integrins are transmembrane heterodimeric glycoproteins composed of two non-covalently bound α and β subunits. Vascular SMC predominantly express fibronectin and collagen-binding integrins which have specific roles in regulating vasoconstriction and cell adhesion [8,9]. Integrin-based cell-matrix adhesions are signaling-rich sites that drive extracellular matrix remodeling and provide vascular SMC with the ability to adapt and distribute cyclic forces caused by the contraction of the heart and blood flow [7,10]. Alteration of the integrin-based adhesions impacts vascular smooth muscle contractility. Any dysfunction occurring in one of the components of this intricate crosstalk mechanism results in impaired structural and functional stability of the aortic wall and causes thoracic aortic diseases such as aortic aneurysms, eventually followed by aortic wall dissection or rupture.

Vascular SMC contractility is primarily driven by smooth muscle α-actin (SMα-actin), which is the predominant actin isoform (65~70%) in SMC, while smooth muscle γ-actin (SMγ-actin) is less present (10~15%) in vascular SMC [10]. Previous studies conducted on aortic SMC isolated from mice lacking *Acta2 (Acta2^−/−^*) [11] showed that aortic SMC have reduced contractility and increased stiffness [12]. We showed that loss of SMα-actin triggers a potential compensatory upregulation of non-dominant SMγ-actin in aortic SMC of *Acta2^−/−^* mice which eventually increases aortic SMC stiffness [2,12]. Additionally, our studies found decreased integrin recruitment at cell-matrix adhesions in *Acta2^−/−^* mice which leads to a decreased ability for mechanosensing and further reduces contractility, leading to thoracic aortic aneurysm and dissection (TAAD) [2]. However, the effect of the heterozygous *ACTA2* p.R149C (*ACTA2^R149C/+^*) variant on integrin-based cell adhesions was not investigated.

Tissue from patients with the heterozygous *ACTA2^R149C/+^* showed hyperplasia of aortic SMC and disoriented elastin fibers when compared to aortic tissue from healthy individuals [5]. Nevertheless, only 60% of carriers have a dissection or require surgical repair of an aneurysm by 70 years of age. *Acta2^R149C/+^* mice share some features common to *Acta2^−/−^* mice and TAAD patients [6]. Aortic SMC isolated from Acta2^R149C/+^ mice exhibit decreased aortic contraction, decreased interaction between SMα-actin and myosin, downregulation of SMα-actin and reduced actin network organization in aortic SMC. Additional experiments found that the downregulation of mutated SMα-actin compared with wild-type SMα-actin in *ACTA2^R149C/+^* cells is due to the retention of mutated SMα-actin in the chaperonin-containing t-complex polypeptide (CCT) which mediates the folding of monomeric actins [6]. Thus, the reduced availability of the mutant SMα-actin in *ACTA2^R149C/+^* cells may delay the thoracic aneurysm formation and decrease the onset of thoracic aortic disease in patients.

The purpose of this study is to investigate the effect of the *Acta2^R149C/+^* variant on actin cytoskeleton architecture, cell adhesion and contractility. We tested the hypothesis that the *Acta2^R149C/+^* variant alters actin isoform expression and decreases integrin recruitment, thus reducing aortic contractility. Our results from *Acta2^R149C/+^* mice suggest that these mice exhibit similar cellular characteristics as *Acta2^−/−^* mice but the functional manifestations are intermediate between *Acta2^−/−^* and wild-type mice. Thus, contractility is reduced in the aorta from *Acta2^R149C/+^*, but to a lesser extent than in *Acta2^−/−^* mice. Similarly, aortic SMC stiffness is increased due to a similar compensatory upregulation of SMγ-actin. In contrast, the reduction in SMα-actin fiber formation in *Acta2^R149C/+^* was significant compared with wild-type (WT) cells, but relatively small with the exception of mutant cells plated on collagen IV. While the formation of α2β1 and α5β1 integrin functional dimers may be somewhat limited in *Acta2^R149C/+^* cells, the strength of cell adhesion to the matrix does not change in *Acta2^R149C/+^* cells in comparison to wild-type cells.

## 2. Results

### 2.1. Functional Impairments in Acta2^R149C/+^ Aorta

**Figure 1:**
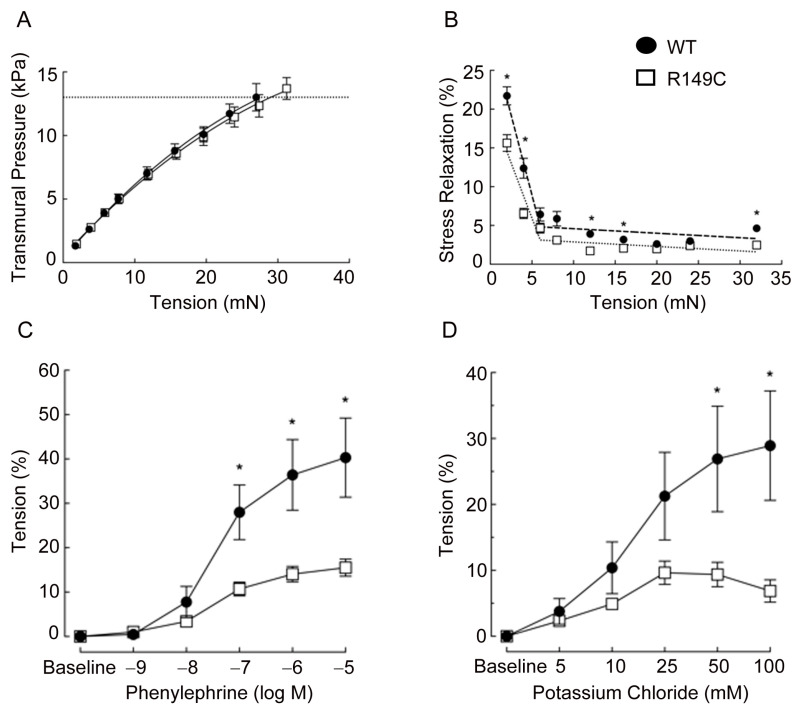
(**A**) Transmural pressure is similar in aorta from *Acta2^R149C/+^* and WT mice. Aortic rings were stretched until a calculated transmural pressure of 13.3 kPa (100 mmHg) was attained (dashed line). (**B**) Stress relaxation was significantly reduced in aorta from *Acta2^R149C/+^* mice compared with WT mice. Thoracic aortic rings were sequentially stretched to elicit 2 mN increases in tension until a calculated transmural pressure of 13.3 kPa was attained. (**C**,**D**) Contractile responses are reduced in aorta from *Acta2^R149C/+^* mice. Cumulative concentration-response curves to (**C**) phenylephrine (PE, 10^−9^ to 10^−5^ M) and (**D**) potassium chloride (KCl, 5–100 mM) were assessed in isolated thoracic aortas from WT and *Acta2^R149C/+^* mice. Cumulative concentration-response curves are expressed as change in tension (%). Values are expressed as mean ± SE. n = 7–8 mice per strain. *, *p* < 0.05 significantly different from *Acta2^R149C/+^*.

A passive length-tension curve was generated from 0 to 32 mN to assess stress relaxation. The maximum was chosen based on calculated transmural pressure reaching 100 mmHg (13.3 kPa). The relation between tension (i.e., stretch) and transmural pressure was similar between the aorta from WT and *Acta2^R149C/+^* mice (Figure 1A). However, stress relaxation showed two functional regimes for aortic tension with a reduction in thoracic aorta from *Acta2^R149C/+^* mice at low tension (Figure 1B) suggesting that overall stiffness is greater in aorta from *Acta2^R149C/+^* mice. Contractile responses to the alpha-1 adrenergic receptor agonist phenylephrine (PE) and receptor-independent potassium chloride (KCl) also were generated to assess contractility. Contractile responses to PE and KCl were significantly reduced in the aorta from *Acta2^R149C/+^* mice. Maximal contractions to PE in *Acta2^R149C/+^* were less than half those of WT (15.5 ± 1.9% vs. 40.3 ± 8.9%, *p* < 0.05) (Figure 1C). Similarly, maximal contractions to KCl in WT were more than double those in *Acta2^R149C/+^* (28.9 ± 8.3% vs. 9.9 ± 1.9%, *p* < 0.05) (Figure 1D). Collectively, these data indicate that contractility is reduced in the aorta from *Acta2^R149C/+^* mice suggesting they are stiffer than those from WT mice.

### 2.2. Smooth Muscle Actin Isoforms Are Differentially Regulated in Acta2^R149C/+^ Aortic Smooth Muscle Cells

**Figure 2:**
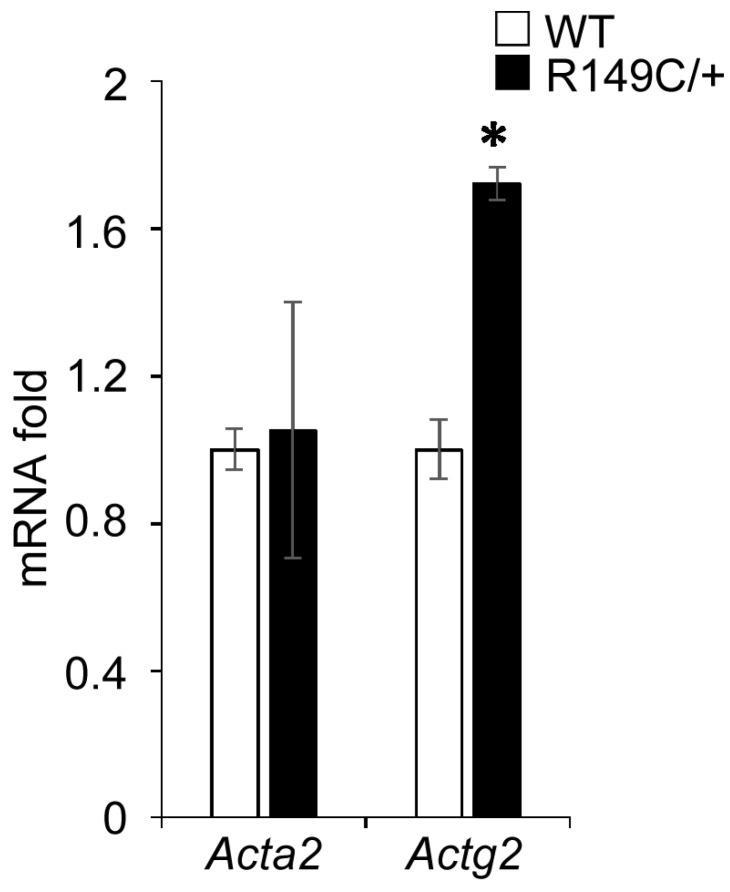
*Actg2* gene expression is elevated in *Acta2^R149C/+^* cells. PCR assays for *Acta2* and *Actg2* were performed on aortic SMC isolated from WT and *Acta2^R149C/+^* mice. Relative mRNA expression levels were normalized to 18S rRNA for each gene. Data are shown as mean ± SD. Significance was evaluated at *p* < 0.05. * Values are significantly different from WT.

**Figure 3:**
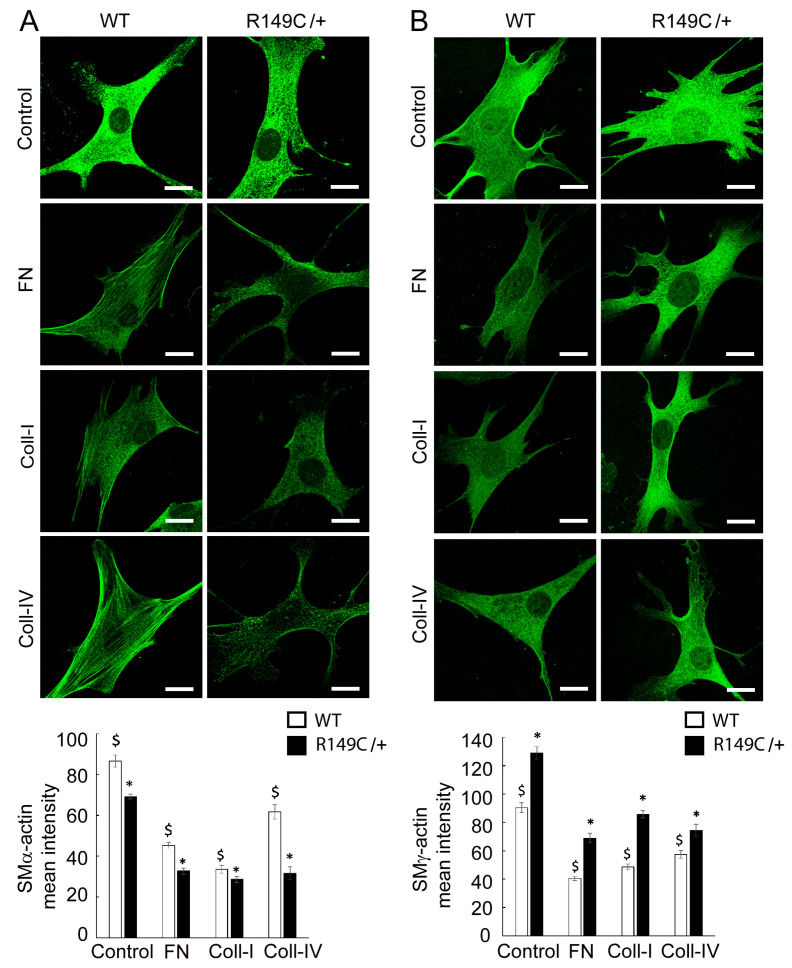
Representative confocal images of (**A**) smooth muscle α-actin (SMα-actin) and (**B**) smooth muscle γ-actin (SMγ-actin) in WT and *Acta2^R149C/+^* cells plated on substrates functionalized with different extracellular matrices. Scale bar represents 20 μm. Quantitative analysis of fluorescence images is shown in the graph (n_α-actin_ = 36–70, n_γ-actin_ = 30–61 cells per condition). Data shown as mean ± SEM. Significance level was considered at *p* < 0.05. * significant difference between WT and *Acta2^R149C/+^*, $ significant difference between WT cells plated on different matrices.

**Figure 4:**
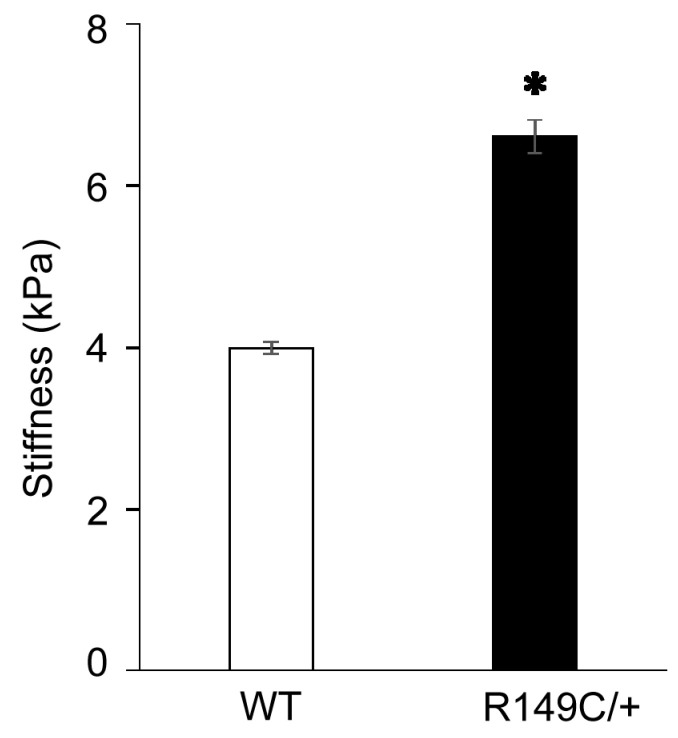
Atomic force microscopy measurements show that cell stiffness is significantly higher in *Acta2^R149C/+^* cells compared to WT cells. Stiffness peak values whose confidence intervals did not overlap were considered significantly different (* *p* < 0.05), n = 2784–2896 individual measurements per condition.

Since our previous studies showed that loss of SMα-actin induces a compensatory increase in SMγ-actin, we asked whether this specific *Acta2^R149C/+^* mutation also affects SMγ-actin expression. Actin mRNA expression was measured in cells isolated from *Acta2^R149C/+^* and WT mice (Figure 2). The transcript level of SMγ-actin was significantly higher in cells isolated from *Acta2^R149C/+^*, whereas there was no difference for SMα-actin. Even though mRNA SMα-actin shows no difference, SMα-actin fiber formation is reduced in *Acta2^R149C/+^* cells. Confocal imaging of mutant vs. WT cells cultured on different extracellular matrices (fibronectin, collagen I, collagen IV) showed a significant decrease in SMα-actin with respect to WT cells over all conditions (Figure 3A), with *Acta2^R149C/+^* showing a similar low level of fluorescence across all matrix-functionalized substrates. In contrast, SMγ-actin presented a much higher level of fluorescence throughout the cytoplasm in the mutant cells compared with WT (Figure 3B). Next, we asked whether these differential changes in actin isoforms affect cell stiffness. Indeed, cell stiffness measurements performed by atomic force microscopy (AFM) showed a significant ~40% increase in stiffness in *Acta2^R149C/+^* cells compared with WT cells (Figure 4).

Taken together, these data suggest that SMα-actin mutation induces a reduced cellular contractility and a compensatory increase in SMγ-actin that could eventually contribute to the increased stiffness of *Acta2^R149C/+^* cells.

### 2.3. Adhesion to the Matrix Is Impaired in Acta2^R149C/+^ Aortic Smooth Muscle Cells

**Figure 5:**
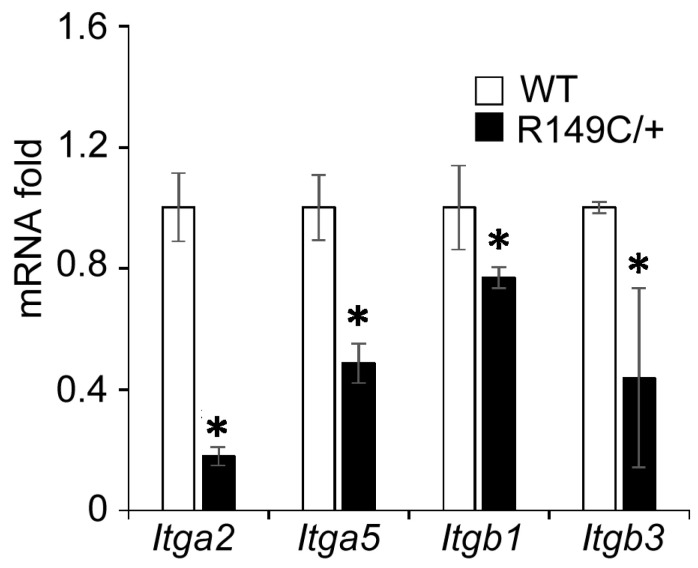
Expression of integrin genes is reduced in *Acta2^R149C/+^* cells. PCR assays were performed on aortic SMC isolated from WT and *Acta2^R149C/+^* mice. Relative mRNA expression levels were normalized to 18S rRNA for each gene. Data are shown as mean ± SD. Significance was evaluated at *p* < 0.05. * Values are significantly different from WT.

**Figure 6:**
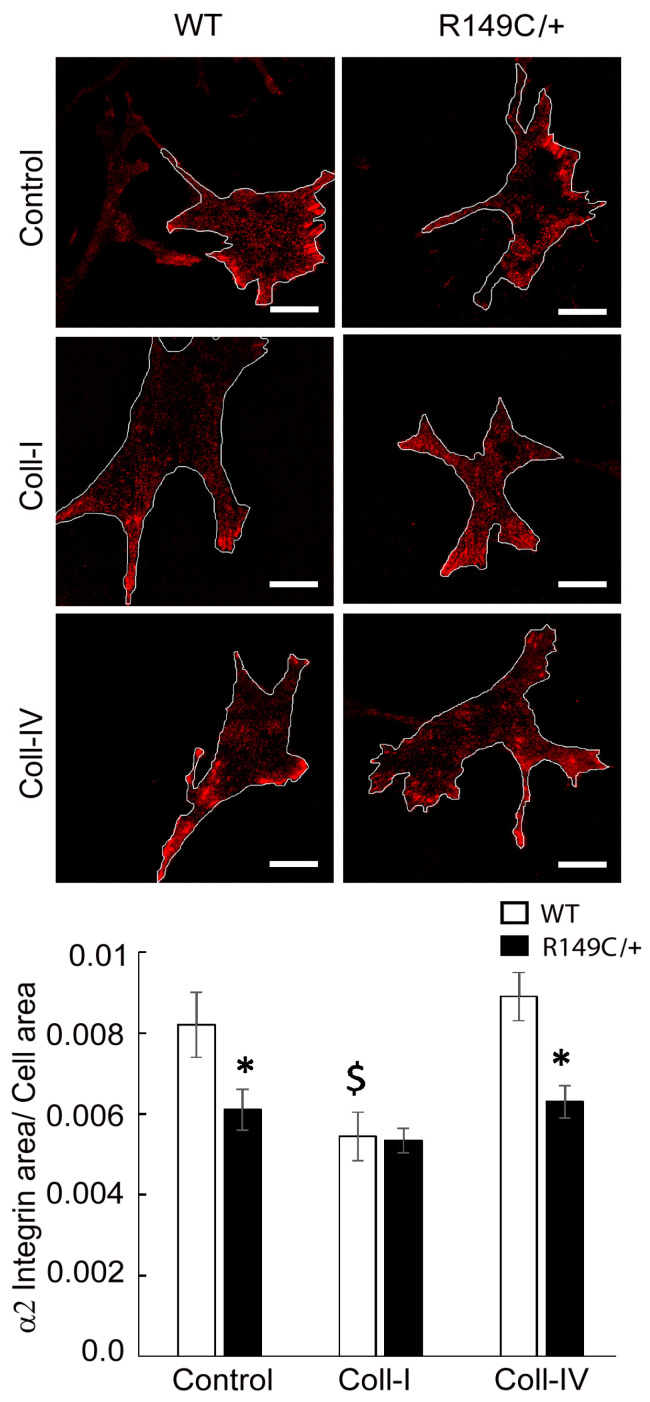
Representative TIRF images of integrin α2 in WT and *Acta2^R149C/+^* cells plated on substrates functionalized with different extracellular matrices. The outer region of the cells was outlined with white lines. Scale bar represents 20 μm. Quantitative analysis of fluorescence images is shown in the graph (n = 32–46 cells per condition). Data shown as mean ± SEM. Significance level was set at *p* < 0.05. * significant difference between WT and *Acta2^R149C/+^*, $ significantly different from WT control and Coll IV plated cells.

**Figure 7:**
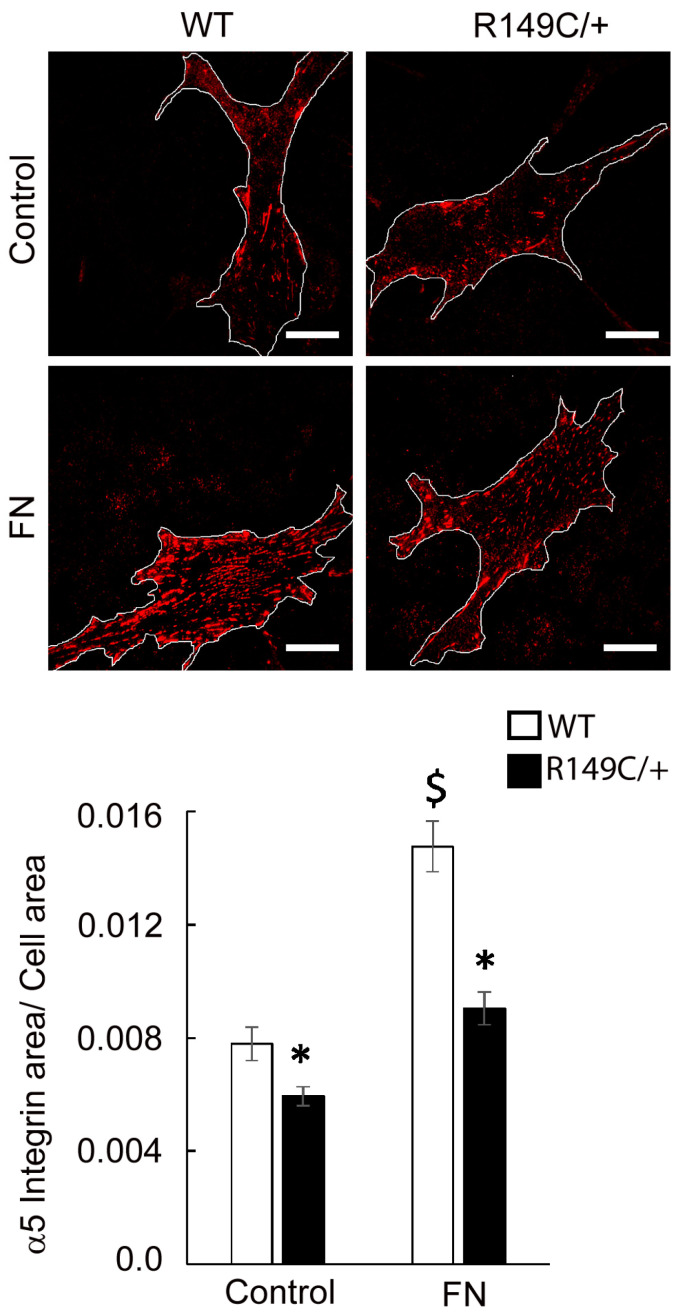
Representative TIRF images of integrin α5 in WT and *Acta2^R149C/+^* cells plated on substrates functionalized with fibronectin or uncoated control substrates. The outer region of the cells was outlined with white lines. Scale bar represents 20 μm. Quantitative analysis of fluorescence images is shown in the graph (n = 46–48 cells per condition). Data shown as mean ± SEM. Significance level was set at *p* < 0.05. * significant difference between WT and *Acta2^R149C/+^*, $ significant difference between WT cells.

Integrin mRNA expression was measured in cells isolated from *Acta2^R149C/+^* and WT mice. Transcript levels of the integrins of interest (α2, α5, β1 and β3) were significantly lower in cells isolated from *Acta2^R149C/+^* mice (Figure 5). Since changes in actomyosin contractility may be associated with an altered mechanosensitive response to external stimuli via cell-matrix crosstalk, we further investigated the effect of extracellular matrix on integrin-mediated cell adhesion. Mutant and WT cells plated on different matrices were immunofluorescently stained for integrin α2, α5, β1 and β3, followed by total internal reflection fluorescence (TIRF) microscopy to characterize the morphology of cell-matrix adhesions. Fluorescence imaging showed that integrin α2 is sporadically present at cell edges with decreased recruitment in *Acta2^R149C/+^* compared with WT cells for both Coll IV and control. No changes were recorded for cells plated on Coll I (Figure 6). Plating cells on fibronectin functionalized substrates elicited a significant increase in integrin α5 recruitment at cell-matrix adhesion sites with a two-fold increase in WT cells by comparison with control, but with a modest increase in *Acta2^R149C/+^* cells (Figure 7). Even though the fibronectin-induced recruitment level was different, integrin α5 showed the same small streak-like pattern with an even distribution across cell basal area for both cell types.

**Figure 8:**
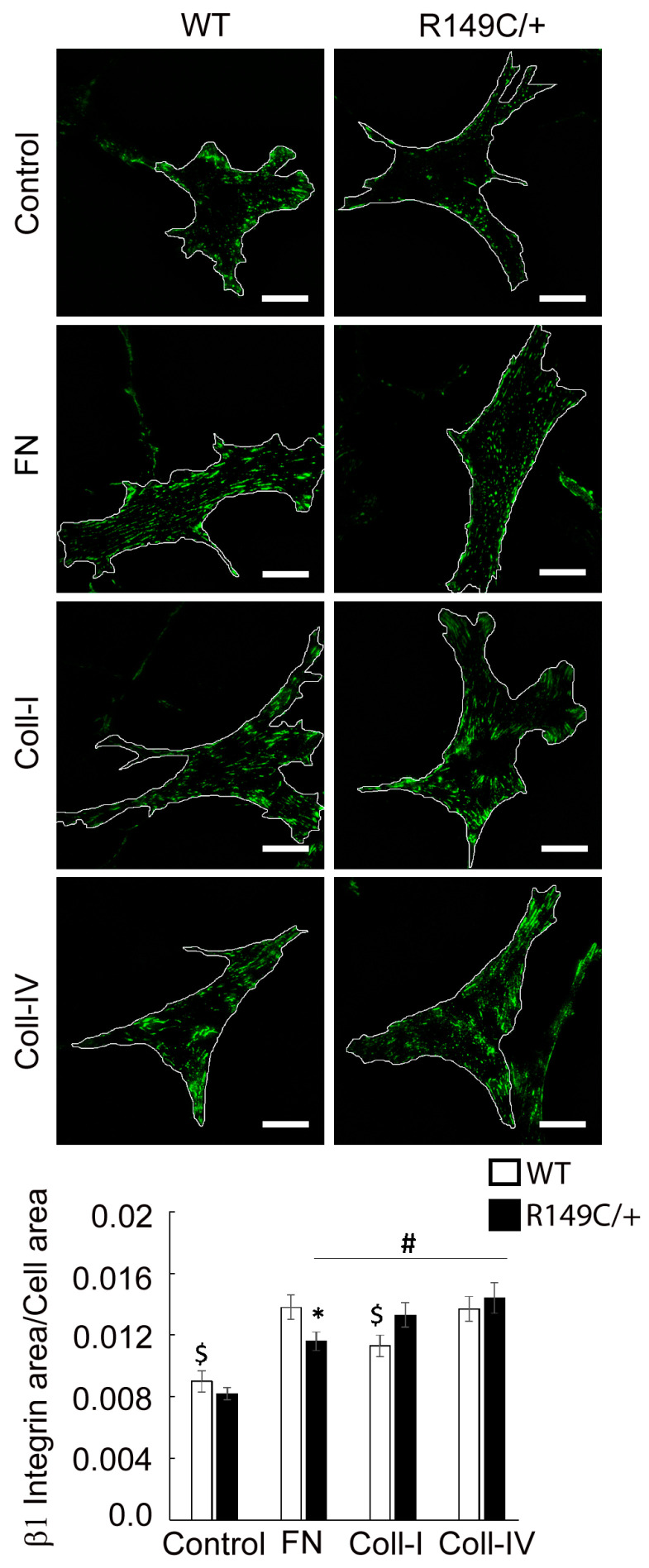
Representative TIRF images of integrin β1 in WT and *Acta2^R149C/+^* cells plated on substrates functionalized with different matrices. The outer region of the cells was outlined with white lines. Scale bar represents 20 μm. Quantitative analysis of fluorescence images is shown in the graph (n = 35–46 cells per condition). Data shown as mean ± SEM. Significance level was set at *p* < 0.05. * significant difference between WT and *Acta2^R149C/+^*, # significant difference between *Acta2^R149C/+^* cells from control, $ significant difference from all WT cells.

**Figure 9:**
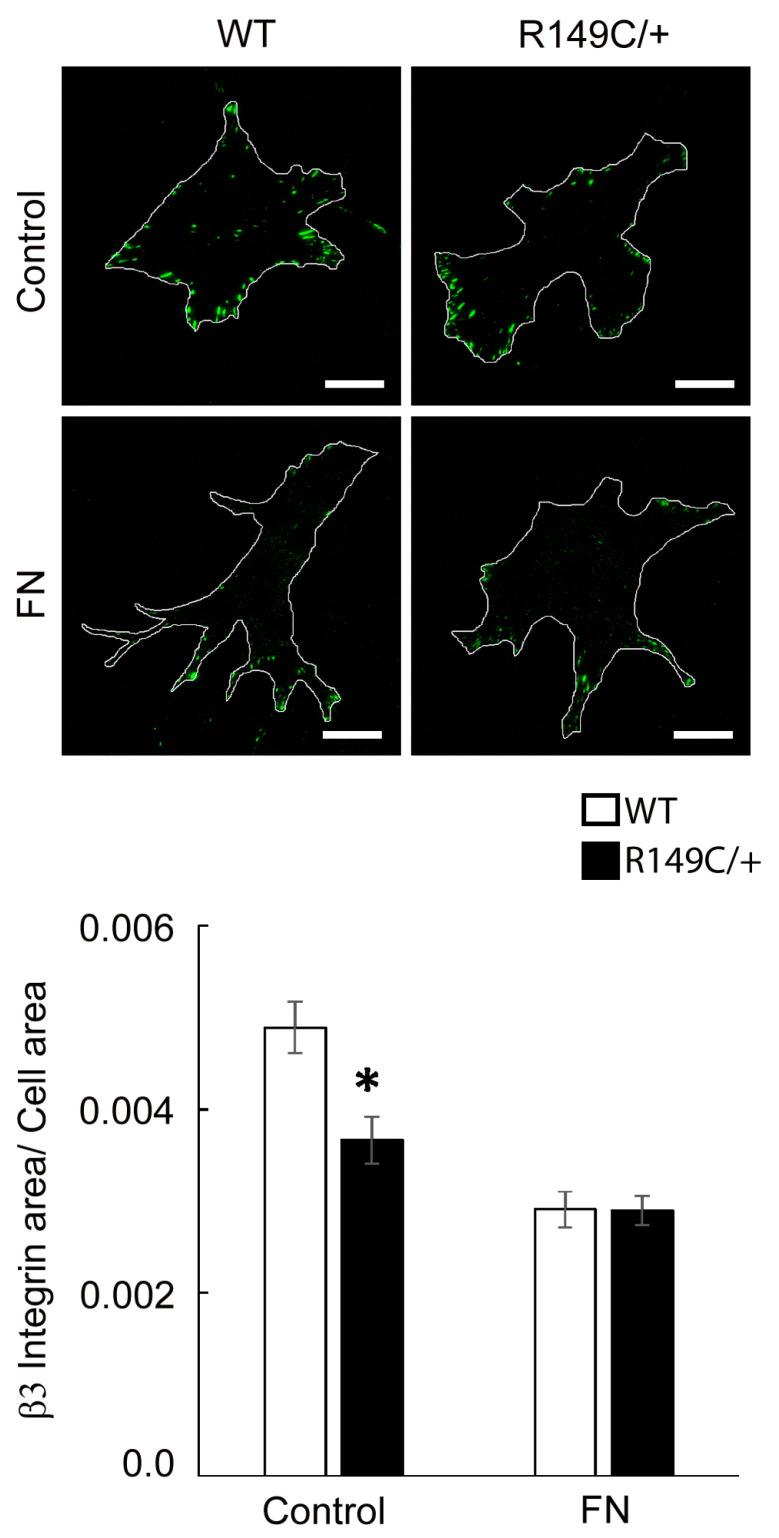
Representative TIRF images of integrin β3 in WT and *Acta2^R149C/+^* cells plated on substrates functionalized with fibronectin or uncoated control substrates. The outer region of the cells was outlined with white lines. Scale bar represents 20 μm. Quantitative analysis of fluorescence images is shown in the graph (n = 35–47 cells per condition). Data shown as mean ± SEM. Significance level was set at *p* < 0.05. * significant difference between WT and *Acta2^R149C/+^* cells.

The integrin β1 subunit pairs with the integrin α5 subunit to bind fibronectin and with the integrin α2 subunit to bind collagen. Our results show that overall β1 integrin expression is also matrix-dependent and significantly higher across all matrices with respect to uncoated, control substrates (Figure 8). Integrin β1 recruitment is slightly decreased in mutant cells plated on fibronectin, while no change was shown between the two cell types for either Coll I and IV. In contrast to the relatively even distribution of integrin β1 throughout the basal cell area, integrin β3 is present in discrete streak-like adhesions only at cell edges, as expected [13]. Quantitative analysis of fluorescence images showed that integrin β3 is significantly reduced in mutant cells with respect to the control, while there is no difference between the cell types plated on fibronectin functionalized substrates (Figure 9).

These data suggest that integrin recruitment is differentially regulated by the matrix, and that integrin α2 and α5 subunits may limit the formation of α2β1 and α5β1 integrin functional dimers in *Acta2^R149C/+^* cells, which is in agreement with reduced integrin expression in these cells (Figure 5). For example, AFM force measurements showed that integrin α5β1 adhesion force to fibronectin was not different in *Acta2^R149C/+^* compared with WT cells (21.94 ± 1.02 pN vs. 21.73 ± 1.43 pN); however, the probability of adhesion that represents the ability of binding free endogenous integrin α5β1 on the apical cell surface decreased ~13%. Taken together, the reduced integrin recruitment in *Acta2^R149C/+^* aortic smooth muscle cells may also lead to a reduced contractile phenotype.

## 3. Discussion

Mutations in *ACTA2*, encoding smooth muscle alpha-actin, are associated with impaired vascular smooth muscle function [14,15]. The most common recurrent variant in *ACTA2* is the arginine 149 to cysteine missense mutation (R149C) which is responsible for approximately 24% of patients who have HTAD [6]. While these patients do not develop aneurysms until late in life, we sought to characterize how this *Acta2^R149C/+^* variant affects the functionality of aortic SMC isolated from *Acta2^R149C/+^* mice. Based on our previous experience with mice lacking SMα-actin (*Acta2^−/−^*), we investigated whether the *Acta2^R149C/+^* mutation would alter actin isoform expression, integrin recruitment, and contractility. We tested the hypothesis that the *Acta2^R149C/+^* variant alters actin isoform expression and decreases integrin recruitment, thus reducing aortic contractility. Our results show that *Acta2^R149C/+^* mice exhibit similar cellular characteristics as *Acta2^−/−^* mice but the functional indicators are intermediate between *Acta2^−/−^* and WT mice. We showed that contractility is reduced in the aorta from *Acta2^R149C/+^* mice, but to a lesser extent than in *Acta2^−/−^* mice. While SMα-actin fiber formation in *Acta2^R149C/+^* cells was somewhat decreased compared with WT cells, a compensatory upregulation of SMγ-actin was induced that, in turn, increased *Acta2^R149C/+^* cell stiffness. Further, we showed that adhesion strength to the matrix did not change in *Acta2^R149C/+^* cells compared with WT cells, but the formation of functional dimers for α2β1 and α5β1 integrins was somewhat limited. Collectively, these results from *Acta2^R149C/+^* mice suggest that functional manifestations of the mutation are moderate relative to *Acta2^−/−^* mice likely due to the retention of mutated SMα-actin in the chaperonin-containing t-complex polypeptide; therefore, less mutated SMα-actin is present in the cytoplasm to participate in actin fiber formation [6].

*Acta2^R149C/+^* mice have increased aortic wall thickness, but reduced cell density [6]. These changes in wall structure imply that the aorta from *Acta2^R149C/+^* mice might have functional impairments. Therefore, we performed functional studies on thoracic aorta from Acta2^R149C/+^ mice to determine the effect of this mutation on the passive and active properties of the aorta. The aorta ring segments were passively stretched until the estimated transmural pressure reached 100 mmHg. There was no difference in transmural pressure between *Acta2^R149C/+^* and WT mice at any level of stretch. This finding is in contrast to the aorta from *Acta2^−/−^* mice which had a reduced transmural pressure [2]. These results imply that *Acta2^R149C/+^* mice produce sufficient wild-type SMα-actin to generate transmural pressure in response to stretch. In addition, we assessed stress relaxation, which is a reduction in tension following a stretch or increase in pressure. Stress relaxation was slightly, but significantly lower in the aorta from *Acta2^R149C/+^* mice. Stress relaxation measurements in thoracic aortic rings showed two functional regimes for aortic tension, with a reduction in thoracic aorta from *Acta2^R149C/+^* mice at low tension values where vessel contractility is predominantly driven by smooth muscle cells. The reduced stress relaxation implies that the aorta in those mice is stiffer than that of WT mice. This increase in stiffness is supported by the finding that aortic SMC from *Acta2^R149C/+^* mice exhibited increased stiffness as measured by AFM (Figure 4). This stiffening may be related to reduced SMα-actin fiber formation that induces a compensatory increase of SMγ-actin in *Acta2^R149C/+^* SMC (Figure 3). The increase in SMγ-actin was also confirmed by qPCR (Figure 2), while the presence of mutant SMα-actin, wild-type SMα-actin and SMγ-actin proteins was previously confirmed [6]. While a similar SMγ-actin compensatory effect was found for aortic SMC lacking SMα-actin, the increase in stiffness in *Acta2^−/−^* cells was three-fold larger compared with WT [12]. Thus, both functional and cell-based measurements point to an intermediate increase in vascular stiffness in the aorta from *Acta2^R149C/+^* mice compared with WT and Acta2^−/−^ mice.

The reduced stress relaxation in the aorta from *Acta2^R149C/+^* mice could impact long-term blood pressure regulation in response to increases in volume. The effect of the increase in vascular stiffness observed in vitro might be offset by the significant decrease in contractility in the aorta from *Acta2^R149C/+^* mice. Responses to PE and KCl in the aorta from *Acta2^R149C/+^* mice were approximately 50% of that observed in the aorta of WT mice. Our results confirm and extend those reported by Chen et al. [6] who described a similar attenuation in contraction in response to maximal concentrations of PE and KCl. In the current study, cumulative concentration-response curves were generated, and contractile responses were decreased at submaximal concentrations of both agents. Therefore, the increases in stiffness that would be expected by smooth muscle activation by PE [16,17] might have been attenuated due to the decreased contractility in the aorta from *Acta2^R149C/+^* mice. Collectively, the decreased stress relaxation and reduced contractile responses to PE are similar to those observed in the aorta from aged (21 mo) C57BL/6 mice [18]. Aorta from old mice also display increased wall thickness and reduced cell density, characteristics also reported for aorta from *Acta2^R149C/+^* mice [6]. Wheeler et al. [18] considered that the phenotype changes observed in the aorta from old mice should predispose them to increased stiffness and potential aortic aneurysm. Interestingly, *Acta2^R149C/+^* mice are normotensive; do not have an increased blood pressure response to transverse aortic constriction or high salt diet plus L-NAME, and hypertensive *Acta2^R149C/+^* mice do not develop aortic aneurysms [6]. It is unclear whether small artery or arteriolar function is altered in *Acta2^R149C/+^* mice. However, the presence of some (i.e., one allele) wild-type SMα-actin appears to be sufficient to regulate vascular tone to prevent aortic disease in *Acta2^R149C/+^* mice in contrast to *Acta2^−/−^* mice that lack SMα-actin and develop aortic aneurysm with age [19].

Vascular SMC are the main players regulating vascular function. However, they are also responsible for secreting and organizing the extracellular matrix that surrounds them. Thus, SMC discrete properties that characterize adhesion to the matrix are important contributors to maintaining vascular tone. Integrins are transmembrane proteins responsible for anchoring the cell cytoskeleton to the matrix via adhesion structures. Our results showed that integrin gene expression levels are reduced in *Acta2^R149C/+^* cells (Figure 5). Integrin α5β1 has an important role in regulating vessel wall contractility [20,21]. Even though integrin α5β1 adhesion strength to the matrix is the same as for WT cells, the reduced recruitment of integrin α5β1 at cell-matrix adhesions supports the reduced contractility of SMC presenting *Acta2^R149C/+^* mutation. Moreover, differential reduction of integrin α2 and β1 recruitment by the collagen matrices for *Acta2^R149C/+^* mutant also modulates how well aortic SMC are anchored into the surrounding extracellular matrix. The anchoring of SMC to elastin fibers has been shown to be defective for *Acta2^−/−^* expressing cells [19]. Integrin αvβ3 binds with higher affinity to fibrillin via the same RGD-binding motif as for fibronectin. Our results showed no difference for fibronectin-induced recruitment of integrin αvβ3 at cell edges for both cell types, thus, suggesting that binding to the elastin unit may not be affected in mutant cells, and may compensate to some extent for the decrease in vascular contractility.

Collectively, these results suggest that the sub-cellular structural elements and overall aorta functionality are modestly affected by the R149C SMα-actin mutation due to its reduced presence in the cellular cytoplasm. Thus, these results suggest that changes in *Acta2^R149C/+^* cells may lead to a moderately reduced contractile phenotype.

## 4. Materials and Methods

### 4.1. Animals

All procedures were approved by the Institutional Animal Care and Use Committee at The University of Texas Health Science Center at Houston and are consistent with the National Institutes of Health guidelines for the care and use of laboratory animals. Heterozygous mice *Acta2^R149C/+^* were generated by introducing an *Arg149Cys* mutation into C57BL/6NJ mice using CRISPR/Cas9 technology as previously described [6]. Mice were allowed ad libitum access to food and water and maintained on a 12 h light:dark cycle (7AM–7PM) in a controlled temperature (21–22 °C). Mice of 8 weeks of age were used for experiments.

### 4.2. Aortic Ring Preparation

Following anesthesia by intraperitoneal injection of Avertin (450 mg/kg), thoracic aortas from *Acta2^R149C/+^* and WT mice were dissected, placed in the ice-cold Hanks’ Balanced Salt Solution, and shipped overnight. Overnight shipment does not affect the passive mechanical properties [2,22]. Once delivered, aortas were cleaned of excessive perivascular tissue and then cut into 2 mm ring segments of equal length under the microscope. Each ring was placed in an organ chamber of a 620M Multi Chamber Myograph System (Danish Myo Technology, Hinnerup, Denmark) filled with 8 mL of oxygenated (95% O_2_, 5% CO_2_) physiological saline solution (118.31 mM NaCl, 4.69 mM KCl, 1.2 mM MgSO_4_, 1.18 mM KH_2_PO_4_, 24.04 mM NaHCO_3_, 0.02 mM EDTA, 2.5 mM CaCl_2_, and 5.5 mM glucose) and allowed to equilibrate at 37 °C for at least 30 min. PSS was maintained at 37 °C and pH 7.4 throughout the experiment.

### 4.3. Stress Relaxation in Aortic Rings

To assess the mechanical properties of the vessel, stress relaxation was assessed in thoracic aortic rings from *Acta2^R149C/+^* and WT mice. Aortic rings were stretched in 2–4 mN increments from 0 mN until the calculated transmural pressure reached 13.3 kPa (100 mmHg) [2]. Transmural pressure was calculated as p = 2 π*T/L, where L is the internal circumference compatible with wall tension T. Length and tension were recorded immediately after each increment of tension and once again after 1 min. Stress relaxation was calculated as the difference between each increase in tension and the tension after 1 min and was expressed as a percent decrease in tension. Passive tension curves were generated by plotting calculated transmural pressure (kPa) versus tension (mN).

### 4.4. Functional Assessment of Contractile Properties

Aortic rings were then stretched to an optional resting tension based on passive stress and relax tension-force assessment [2]. Cumulative concentration-response curves to phenylephrine (PE, 10^−9^ to 10^−5^ M) and potassium chloride (KCl, 5–100 mM) were generated to assess contractile function. Doses were added after the response curve reached a plateau from the previous dose. Percent vasocontractile responses (%) were calculated for PE and KCl as [(*D_P_* − *D_B_*)/*D_B_*] × 100, where ‘*D_P_*’ is the maximal force generated by a given specific dose and ‘*D_B_*’ is the baseline force.

### 4.5. Smooth Muscle Cell Culture

Aortic SMC were explanted from the ascending aorta from male *Acta2^R149C/+^* and WT littermates as previously described [23]. Cells were cultured in Smooth Muscle Basal Medium (PromoCell, Heidelberg, Germany) supplemented with 20% fetal bovine serum, 20 mM HEPES (Sigma-Aldrich, Saint Louis, MO, USA), 2 mM L-glutamine, 1 mM sodium pyruvate, 100 U/mL penicillin, 100 mg/mL streptomycin, and 0.25 mg/mL amphotericin B. Cell cultures were maintained in an incubator at 37 °C with 5% CO_2_. For experiments, cells were then plated on 35 mm glass bottom dishes (MatTek, Ashland, MA, USA) functionalized with 20 μg/mL of fibronectin (FN) (Sigma, Saint Louis, MO, USA), collagen I (Coll I) (Sigma, Saint Louis, MO, USA) or collagen IV (Coll IV) (Millipore, Billerica, MA, USA) as previously described [2]. Cells plated on uncoated substrates were used as controls. Five hours after plating, cells were starved overnight in the same cell culture media with 1% FBS and without growth factors. All reagents were purchased from Invitrogen (Carlsbad, CA, USA), unless otherwise specified.

### 4.6. Quantitative RT-PCR

Aortic SMC isolated from *Acta2^R149C/+^* and WT mice were subjected to total RNA extraction. Briefly, 100,000 cells were seeded on uncoated six-well plates in complete cell culture medium and incubated for 72 h. RNA was isolated using the PureLink RNA Mini kit (ThermoFisher Scientific, Waltham, MA, USA) and quantified using Nanodrop (ThermoFisher Scientific, Waltham, MA, USA). cDNA was synthesized from the isolated RNA using QScript reagent (Quantabio, Beverly, MA, USA) and qRT-PCR was performed using SYBR Green chemistry with PerfeCTa SYBR^®^ Green FastMix (Quantabio, Beverly, MA, USA). The expression of all genes tested was normalized to 18S rRNA. Detailed information on the primer sequences (Millipore Sigma, Burlington, MA, USA) is presented in Table 1.

### 4.7. Immunofluorescence Staining

Aortic SMC were fixed at 24 hrs after plating by immersion in 2% paraformaldehyde in Dulbecco’s phosphate buffered saline (DPBS). Cells were then washed in a glycine buffer and incubated overnight at 4 °C with primary antibodies against integrin α2 (Abcam, San Francisco, CA, USA), integrin α5 (Milipore Sigma, Burlington, MA, USA), and smooth muscle α-actin (SMα-actin) (Millipore Sigma, Burlington, MA, USA) diluted in a sodium citrate buffer containing BSA and Triton X [24]. After washing, cells were incubated with Alexa 568 secondary antibody (Invitrogen, Carlsbad, CA, USA) for 1 h at room temperature, washed again and immediately imaged in DPBS. A similar procedure with overnight incubation was followed for the primary antibody against integrin β1 or β3 both pre-conjugated with Alexa 488 (BioLegends, San Diego, CA, USA). For smooth muscle γ-actin (SMγ-actin, *Actg2*) staining, cells were first fixed with 1% paraformaldehyde in DPBS followed by permeabilization with cold methanol [25]. Staining was performed as described by using an SMγ-actin primary antibody [26,27] followed by Alexa 488 secondary antibody (Jackson Immuno Research, West Grove, PA, USA).

### 4.8. Smooth Muscle Cell Imaging

Total internal reflection fluorescence (TIRF) microscopy was used to image integrins at cell-matrix adhesions as previously described [28]. Briefly, TIRF imaging was performed using an Olympus IX81 microscope (Olympus, Tokyo, Japan) equipped with a PLAN APO 60X oil 1.45 NA objective lens and a CoolSnap HQ camera from Teledyne Photometrics (Tucson, AZ, USA) using an exposure time of 100 ms [29]. Confocal fluorescence microscopy was used to image the actin isoforms throughout the cell body. An Olympus Fluoview FV3000 Confocal Microscope equipped with UPLSAPO 60XS immersion oil 1.25 NA objective lens was used to acquire 3D stacks of 10–14 planes at 0.25 µm step size with 100 ms exposure time. Images were further analyzed and presented as XY projections. Single-cell imaging was carried out using the same experimental parameters for each data set, for both TIRF and confocal microscopy, respectively.

### 4.9. Fluorescence Image Analysis

To quantify specific protein fluorescence, masking tools and image statistics tools in Slidebook software 6.0 (Intelligent Imaging Innovations, Denver, CO, USA) were used [30]. Briefly, TIRF images were used to quantify integrins at cell-matrix adhesions, while XY projections of confocal images were used to quantify the actin cytoskeleton. Since we compare a large number of cells for each condition, the fluorescence protein area, representing the relative protein density at the specific sites [31], was normalized to the total cell area for each cell before statistical analysis.

### 4.10. Statistical Analysis

#### 4.10.1. Aortic Ring Measurements

Analysis of variance (ANOVA) followed by Bonferroni posthoc analysis was used to assess strain differences for stress relaxation, passive tension curves, and contractile responses to PE and KCl. All values are presented as mean ± SEM (standard error of the mean). Statistical significance was evaluated at *p* < 0.05. Statistical analysis was carried out using GraphPad Prism 9.0 and JMP Pro 16 software.

#### 4.10.2. Fluorescence Measurements

The normality assumption was checked using Q-Q plot and Shapiro–Wilk test. Multivariable linear regression was implemented and estimated model coefficients with 95% confidence intervals were reported. Statistical significance was reported if the significance level alpha was less than or equal to 0.05 (*p* ≤ 0.05). Statistical analysis was carried out using STATA software 16.0 (Stata LLC, College Station, TX, USA).

### 4.11. Atomic Force Microscopy Measurements

Atomic Force Microscopy (AFM) measurements were performed on live *Acta2^R149C/+^* and WT cells in cell culture medium by using MLCT-Bio probes (Bruker Nano Surfaces Inc., Santa Barbara, CA, USA) with a spring constant of 12.2 ± 0.4 pN/nm. The step-by-step process for functionalizing the probes with fibronectin (1 mg/mL, Thermo Fisher Scientific, Waltham, MA, USA) was previously described [32]. The probe was set to approach and retract from the surface of a cell at 800 nm/s. Experiments were performed in triplicates. A total of n = 2700–3200 individual force curves were analyzed for each experimental condition. The adhesion force and cell stiffness at the point of contact were calculated using NForceR software [33], followed by PeakFit 4.11 software (Systat Software Inc., San Jose CA, USA) to estimate the confidence intervals for each distribution. Peaks were considered significantly different (*p* < 0.05) if their confidence intervals did not overlap [34]. The number of adhesions was expressed as a percentage of total events, based on the number of adhesion and non-adhesion events [35].

## Figures and Tables

**Table 1:** Primer sequencies used for qRT-PCR.

Gene	Forward (5′ -> 3′)	Reverse (5′ -> 3′)
*Acta2*	GTCCCAGACATCAGGGAGTAA	TCGGATACTTCAGCGTCAGGA
*Actg2*	CCGCCCTAGACATCAGGGT	TCTTCTGGTGCTACTCGAAGC
*Itga2*	TACAGACGTGCTCCTGGTAGGT	CCGAGCATTTCCAGTGCCTTCT
*Itga5*	GTGTGAGGAACTGGTCGCCTAT	CCGTTCTCTGGTCCAACCGATA
*Itgb1*	CTCCAGAAGGTGGCTTTGATGC	GTGAAACCCAGCATCCGTGGAA
*Itgb3*	GTGAGTGCGATGACTTCTCCTG	CAGGTGTCAGTGCGTGTAGTAC
*18S*	GTAACCCGTTGAACCCCATT	CCATCCAATCGGTAGTAGCG

## Data Availability

The data presented in this study are available upon request from the corresponding author.
